# Development of a Lived Experience-Based Digital Resource for a Digitally-Assisted Peer Support Program for Young People Experiencing Psychosis

**DOI:** 10.3389/fpsyt.2020.00635

**Published:** 2020-07-02

**Authors:** Claire E. Peck, Michelle H. Lim, Melanie Purkiss, Fiona Foley, Liza Hopkins, Neil Thomas

**Affiliations:** ^1^ Centre for Mental Health, Swinburne University of Technology, Melbourne, VIC, Australia; ^2^ Iverson Health Innovation Research Institute, Swinburne University of Technology, Melbourne, VIC, Australia; ^3^ Headspace Youth Early Psychosis Program, Alfred Health, Melbourne, VIC, Australia; ^4^ Alfred Mental and Addiction Health, Alfred Hospital, Melbourne, VIC, Australia

**Keywords:** peer support, first episode psychosis, personal recovery, digital mental health, early intervention mental health services

## Abstract

This paper describes the creation of a web-based digital resource designed for tablet computer use during peer work sessions to structure discussion about recovery in early psychosis. The resource consisted of a series of videos featuring young people who have used early psychosis services discussing how they navigated issues in their own recovery. A participatory process was used to create the resource. Researchers held a series of collaborative development workshops with early psychosis service users, peer workers, other mental health practitioners, and academics. These were used to derive a framework of recovery processes relevant to young people experiencing psychosis, which was considered as useful areas of discussion within a peer work relationship. A semi-structured interview guide based on this framework was then used in video-recorded interviews with young people in recovery from psychosis. Footage was edited into 14 videos and organized into six final themes: My Journey, Self-Care, Connections, My Identity, Life, and Mental Health. The combined expertise of young mental health service users, peer support workers, mental health practitioners, and digital mental health researchers throughout the development process enabled the creation of tailored digital resource for peer work in an early psychosis service.

## Introduction

Early intervention for young people experiencing psychosis has become a key focus of mental health services worldwide (1). Policies such as the Australian Government’s National Youth Mental Health Initiative have led to widespread implementation of specialized early intervention mental health services for young people experiencing or at risk of psychosis (1–3). Engaging young people with psychosis into mental health services earlier in their mental health journey has been reported to improve clinical and psychosocial outcomes and reduce the long-term burden of mental illness (4–6).

Mental health services have been shifting towards a recovery-oriented approach to mental health treatment. This has been heavily influenced by a broader consumer movement in mental health advocating for mental health services to prioritize personally meaningful goals of consumers, rather than narrowly focussing on symptom remission (7, 8). This focus of services is often referred to as *personal recovery* to distinguish it from traditional clinical (symptom remission) and functional (return to work or study) conceptualizations of recovery. This concept of personal recovery emphasizes a process whereby individuals live a hopeful, rewarding, and satisfying life, sometimes in the presence of ongoing symptoms (7, 9, 10). In a synthesis of the existing literature, Leamy et al. (11) proposed a framework of five personal recovery themes, highlighted from mental health consumer accounts of their own recovery from severe mental illness. These include: social connectedness; hope and optimism about the future; transforming identity; finding meaning and purpose; and empowerment in mental health self-management (CHIME) (11). CHIME has been cited as a model for defining and examining personal recovery in mental health, and may have benefits in providing an evidence-based approach to help inform clinical and research programs (12–15). Although, criticisms have been raised in regard to the model having an optimistic perspective on recovery and under-emphasising the difficulties associated with mental illness (16–19). The CHIME model was also conceptualized from long-term service users lived experiences, in which the relatedness of the model to younger populations, including those experiencing early psychosis is uncertain.

In supporting the notion of recovery, an emerging feature of recovery-oriented mental health services is engaging people with lived experience of mental health difficulties into service delivery (7, 20, 21). Peer Support Workers (PSWs) are employed by mental health services and often use their personal experiences of mental illness, “along with relevant training and supervision to facilitate, guide, and mentor another person’s recovery journey by instilling hope, modeling recovery, and supporting people in their own efforts to reclaim meaningful and gratifying lives in the communities of their choice” [p. 4, (22)]. The shared lived experience of mental illness can help create a sense of authenticity, trust, understanding, acceptance and support in developing adaptive self-management strategies, counteracting negative stereotypes of mental illness, and making it evident that recovery is possible (21, 23–26).

To date, resources to facilitate the delivery of peer work have most commonly been created in the form of paper workbooks [e.g., Wellness Recovery Action Planning; (27)]. There may be benefit in using digital tools in sessions to help structure the peer work process, particularly in young people experiencing psychosis (28–30). The Self-Management and Recovery Technology (SMART) research program in Victoria, Australia has been examining the use of digital lived experience material on tablet computers (31–33). Given that recovery concepts have arisen from mental health consumer narratives of recovery, the resource developed in the SMART research program makes use of lived experience videos as a means of communicating content. Videos relate to a series of recovery topics, each feature a number of “peers” discussing how that issue has affected them and/or how they have navigated it (31, 32). Initial findings confirm that use of lived experience video material is a feasible and acceptable means of structuring one-to-one sessions (31, 34). Participants described the videos providing a sense of hope and connection, encouraged participants to talk about their own mental health, and gained a sense of ownership over the conversations discussed (34). These findings draw similarities with naturalistic explorations of peer videos on social media and digital storytelling (35–37).

This novel form of working has potential synergies with a peer work context. Peer workers may be better placed than non-peer workers to discuss the lived experience-based material. Meanwhile, the lived experience video material may be useful both in providing a reference point for the PSW sharing parts of their own lived experience, and in providing a broader range of lived experience material to discuss than their own alone to promote recovery-oriented discussions. The increased availability and diversity of peer lived-experience through videos may also help improve participant feelings of normalization, validation, and hope.

Preliminary investigations indicate that young people with psychosis have expressed interest in digital resources including videos being used as part of their mental health service support (28, 29, 38). Bucci et al. (38) explored the views of young people with psychosis on digital health interventions, finding that digital interventions were seen as an acceptable resource that could aid in destigmatizing access to mental health services. Current examples of digital mental health interventions for young people experiencing psychosis include HORYZONS—an online program that contains a series of interactive psychosocial interventions and an expert and peer social networking platform (39). Investigations into HORYZONS have found the intervention was feasible, with participants viewing the online intervention as being positive and useful, and feeling more empowered and connected (39). Bucci and colleagues (40) trial of a digital smartphone application (i.e., Actissist) designed to target key areas of concern for early psychosis was found to be a feasible, acceptable, and safe digital intervention. Further research has provided support for the feasibility and acceptability of digital programs in this population (41–46), with a few randomized control trials currently under-investigation (47, 48).

However, the development of digital resources to promote personal recovery in young people experiencing psychosis, and their use in a peer work context are yet to be examined. In developing such a resource, the limited available literature on personal recovery and individual face-to-face peer support work in this population emphasizes the key role that participatory processes can have. Consumer expertise has been increasingly encouraged within health research by government funders such as the US National Institute of Mental Health (49), Australian National Health and Medical Research Council (50), and UK National Institute of Health Research (51). Likewise, participatory design processes are increasingly becoming the standard in digital intervention development (52–54), and in mental health service design (55).

Meaningfully integrating expertise from consumers and mental health service professionals including PSWs *via* a participatory process is needed to create resources that are more likely to align with the needs and preferences of those involved in delivering and receiving the program (56–59). It may also assist in understanding how digital technology can be integrated into peer work and help break down some of the barriers often associated with implementing peer-based programs (29, 60, 61). In this paper, we describe the participatory development process we used for making personal recovery lived experience video resources for use in peer support work, and detail the resultant web-based resource. The strengths and difficulties associated with this process will also be discussed.

## Methods

### Development Process

#### Service Context

The project took place in collaboration with Alfred headspace Youth Early Psychosis Program (hYEPP) clinicians, PSWs, and consumers. This service forms part of a nationally-implemented model of services designed for young people aged 12 to 25 years old who are experiencing or at risk of developing psychosis. hYEPP provides the opportunity for young people and their network to receive a holistic approach towards treatment that includes addressing concerns related to psychosis, whilst also supporting young people in key developmental areas such as physical health, education, employment, and relationships (2, 3). The collaboration coincided with the service exploring options for their newly developed peer workforce. This led to the aim of exploring a novel digitally assisted peer support program influenced by work on developing a tablet computer-based recovery-oriented intervention within adult mental health services (31–34). The project was approved by The Alfred (526/16) and Swinburne University of Technology (2018/164) Human Research Ethics Committees. All participants provided informed consent prior to participation.

#### Overview of the Development Process

**Figure 1:**
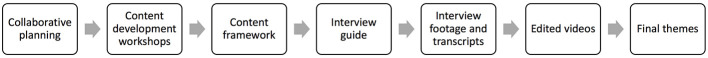
Outline of the development process.

The creation of the resource content followed a sequential development process, involving participatory development of an initial content framework of themes, which was used to develop a semi-structured interview guide, conducting filmed interviews on the basis of this guide, and then editing video material with reference to the initial content framework into final themes (**Figure 1**). In this way, the content was shaped by both participatory development of an initial lived experience-based framework, and then the emergence of lived experience-based material during interviews.

### Co-Development of Content Framework

#### Step 1. Collaborative planning

Initially, a steering group was formed of academics with experience in digital resource development, hYEPP service managers and PSWs to consider the potential use of a tablet-based lived experience video resource within a peer work context.

Discussion over a number of planning sessions involved thinking about the type of peer-resource that could be developed and exploring the principles and experiential nature of peer work and meaning of personal recovery for young people. These discussions highlighted the need to identify the personal recovery priorities that may be relevant for young people with psychosis. They also highlighted the potential for facilitating participatory engagement *via* the “Discovery College” program, a youth-oriented version of the recovery college model, an innovation in recovery-oriented practice with an emphasis on collaborative learning processes in mental health self-management (62).

#### Step 2. Participatory Content Development Workshops

Four, 3 h consumer workshops were co-designed, developed, and conducted in 2016. The workshops were facilitated by two members of the hYEPP Discovery College team, with members of the steering group present. Use of independent facilitators helped to reduce the potential biasing of the emergent content framework towards a replication of SMART or CHIME. Any PSW or service user engaged with the service was eligible to participate in the content development workshops. Two PSWs (both female) and ten hYEPP service users (3 males and 7 females) participated.

In the first workshop, participants were oriented to the type of digitally assisted peer program that could be created and discussions explored young people’s perspectives on the relevance of content and videos (e.g., lived experience and professional videos) from the SMART resources developed for a mainstream adult population (31). Discussion initially involved an introduction to participatory research and peer support work to help orient participants to the purpose of the workshops. The group was then asked to consider a young person who experiences mental health challenges and may have started to use mental health services like this one for the first time and using a digital resource with a peer worker. They were asked questions such as “what is important to know and understand about their mental health and wellbeing?”, “what is important to have in their lives?”, “what is important to do to stay well?”, and “what is important to include in a digital resource for young people experiencing mental health challenges?”. From these discussions, overall themes emerged. The second and third workshops focussed on determining the specific information to include within each of the themes identified and creating interview questions for the lived-experience filming in order to capture the central target of each theme. In the workshops, participants also decided on a name for each area that they felt was relevant to a youth population and discussed their perspectives on the development of a website to host the program (e.g., use of color and youth-friendly language).

### Initial Themes From the Content Development Workshops

Themes elicited revealed several areas within the individual and their environment as being important to their recovery. For example, participants expressed a preference for hearing other young people’s perspectives on family pressures or difficult parent situations and how these situations were navigated. Participants also advocated for content focussing on de-mystifying the mental health sector and navigating engagement with mental health services, such as how to break down barriers around diagnosis and medications and discuss concerns with workers. Environmental factors such as conversations about managing school, driving, and finances were reported, as well as making conscious choices around social media use. A key theme that arose across each of the personal recovery areas was the importance of creating authentic and genuine lived experience accounts that capture hopeful stories of people doing well but also include “not all good stories” to normalize the challenges experienced.

Six personal recovery themes were summarized; 1) recovery, 2) relationships, 3) self-management, 4) basic needs/environment, 5) identity, and 6) resources/stigma. The themes were renamed following a detailed discussion of each area to capture the overall content and to create titles that would be in appropriate language for a youth population. For instance, basic needs/environment was re-titled “Life” to encompass the different life challenges identified by young people. Recovery was re-titled “My Journey” because participants expressed dislike for the term “recovery”, as it implied there is an end-point to when someone has “recovered’” from mental illness.

### Conducting the Lived Experience Filming

#### Step 3. Development of Semi-Structured Interview Schedule

**Figure 2:**
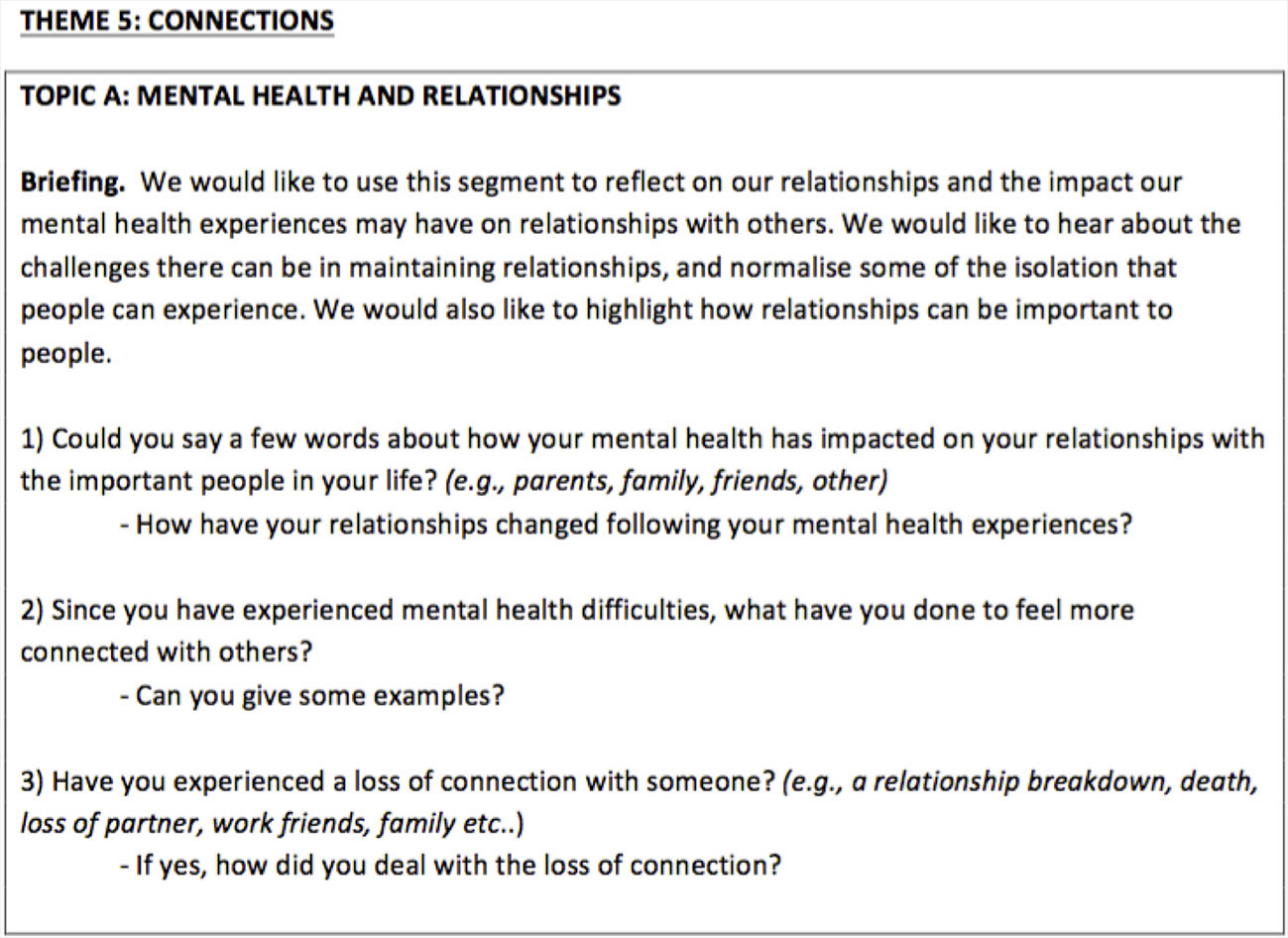
Example of the lived experience filming interview schedule.

In the next step, a semi-structured interview schedule was developed based on the list of interview questions suggested by participants for each theme in the content development workshops by CP, FF, and NT. Open questions were developed to elicit the interviewees’ experiences of each of the themes. An example of the interview schedule is shown in **Figure 2**.

#### Step 4. Recruitment and Briefing of Interviewees

Advertisements were co-designed and created with the service to identify young people aged 18 to 25 years old who had an experience of psychosis and were willing to share their lived experience of mental health on film. Recruitment involved distributing flyers at the service, liaison with mental health clinicians, and discussions with service users who were involved in analogous events. Interested individuals were contacted to discuss the nature of their participation and potential implications of the filming. A pool of interviewees was sought to capture diversity in gender, age, sexuality, and ethnicity.

Ten young adults (4 females, 6 males) aged 18 to 31 years old (*M =* 23.10, *SD =* 3.84) with an experience of psychosis were recruited to be filmed and interviewed about their recovery. Two of the 10 young adults’ footage was included from another resource [i.e., (31)] due to difficulty experienced in recruitment. Participants were emailed the consent form and interview questions one week prior to the scheduled filming. This allowed for the participant to review the consent form and the interview questions and inform the researchers of any areas they would not like to discuss during the filming. This was revisited on the day of the filming to confirm the participant would only be asked questions they were comfortable with.

#### Step 5. Interviewing and Filming

Interviews with young people were then conducted by CP, NT, FF, and/or ML across a total of four days in 2016 and 2017, using a professional videographer team to film the interviews. NT, FF, and ML had experience in conducting lived experience interviews. A two-camera set up was used, with the interviewee in frame of both cameras, and the interviewer outside the camera’s field of view. The use of two cameras allowed for switching view from one camera view to another when material need to be edited out to create a seamless edit. Furniture and other styling items were brought to the filming to create diverse background scenes. Audio was recorded using a lapel microphone.

During the interview, the interviewer asked open questions focused on participants experiences and explored how they navigated various challenges, based on the semi-structured interview schedule. The format allowed for the interviewer to deviate from the schedule, where it appeared useful to explore additional emerging content. Interviewees were briefed to discuss their own experiences and encouraged to use a first-person perspective (e.g. “something I experienced has been…”, “I’ve found…”, or “for me…”, rather than “people with psychosis experience…” or “if you use mental health services you should…”). This approach was used to fit with the theme of lived experience and capture the uniqueness of individuals’ experiences and perspectives. Participants were asked to re-film their response to improve delivery or align with the first-person perspective, if required. Each interview went for approximately 1–1.5 h and participants were financially reimbursed with $200 (AUD). The interviews were professionally transcribed, and a two-week cooling off-period was provided for participants to review the video transcripts and inform researchers of any material they wished not to be included in the final videos.

#### Step 6. Editing

An extensive editing process was employed to create the lived experience videos. The video transcripts were initially reviewed by CP and coded into the personal recovery themes identified in the consumer content development workshops. Comments were made on the transcripts to highlight content that: i) conveyed a meaningful or powerful message, ii) was relevant to the personal recovery themes, and iii) aligned with the intended purpose of the video. Participant transcripts were cross-checked with the video material to ensure content maintained a similar meaning when visually-presented. Each participant video was then reviewed to identify any visually-presented material that conveyed a meaningful message but may not have appeared important when looking at the participant transcript independently. These steps were completed a total of three times for a thorough review of the material.

Following this, 16 draft videos were created using Camtasia Version 2.10.8 software program (63). The *a priori* parameters for each video were to be less than 4 min in duration (ideally between 2 and 3 min), and to feature at least three interviewees in order to provide diversity in perspectives (important given the highly individual nature of personal recovery) (11). To generate these videos, excerpts from each interviewee’s highlighted content were combined into a video compilation to compare, contrast, and review to determine which material should be retained for the final videos. In reviewing the draft videos, the meaning, significance and range of content, diversity of demographics, and duration of the video were considered to decide on the final content. This was an iterative process reviewed initially by CP, FF, ML, and NT and then with hYEPP PSWs and other team members for feedback and modifications. The final videos were developed by a professional film team. After the videos were created, interviewees involved in the filming were given the option of attending a session to view the finished videos at the mental health service (which two participants took up).

## Results

### Developed Resource

The methodological descriptions outlined the processes involved in creating a lived experienced-based resource for PSWs to use in individual face-to-face sessions with young people engaged in an early intervention mental health service. The results section will commence by detailing the final personal recovery themes developed, then the lived experience videos and website created and, end with describing the resultant digitally assisted peer support program, namely, Peer Plus.

### Final Themes Developed

Through reviewing the content obtained from the lived experience filming, the initial six themes were refined. The filmed interviews were able to elicit material for each of these six themes, but slight modifications were made, where necessary, to ensure each personal recovery theme reflected relevant experiences. For example, the Mental Health theme had been initially designed to have a single video focused on experiences and difficulties associated with medication, but this was incorporated into a video on people’s experiences navigating challenges with mental health services, allowing for a separate video to be created that focussed on people’s experiences with mental health services (both positive and negative).

Overall, six themes emerged from the consultation with the existing literature, lived experience expertise from the content development workshops and film content, and mental health service including PSWs. In the digitally assisted peer program, each personal recovery theme includes one to three lived experience videos and represents a single module within the program. A summary of the six modules is detailed below:

1. *My Journey*, provides an overview of the lived experience speaker’s mental health journey and their view on the meaning of recovery.
2. *Self-Care*, explores how stress affects people including the types of things that cause people stress, how to recognize the signs of being under stress, and strategies to cope with stress.
3. *My Identity,* explores people’s sense of self and how personal identity may be affected by mental illness and the stigma that can be attached to mental health. It includes discussions about how to navigate changes to our personal identity and coping with stigma or discrimination in order to lead a more meaningful and satisfying life.
4. *Connections,* explores the impact of mental health on relationships with friends, family, people in the community and on social media. This includes how these relationships can be challenged during hard times, and ways to nurture these relationships and form new connections with others.
5. *Life*, explores the different challenges people may experience during their late adolescence and young adulthood, and the ways people can navigate these challenges (e.g., getting a driver’s license whilst on medication). Life also covers people’s experiences with disclosing their mental health to others, or factors considered when deciding not to disclose their mental health.
6. *Mental Health,* explores people’s experiences with mental health services and how people could navigate difficulties (e.g., conversations about medication) to make the most of their mental health service.

### Content of Lived Experience Videos

Although initially aiming to produce 16 videos on the basis of funding and content derived from the workshops, a decision was made to have a total of 14 videos (see **Table 1**). There was insufficient content obtained for the My Journey theme, which resulted in one of the videos being removed that focused on participants current situation and future plans. A second video was removed from the Life theme, with two of the planned videos collapsing into a single video to provide an overall perspective on people’s experiences and navigation of difficulties. The final videos are from 1.30 to 3.53 min (*M* = 2.70 min) in duration and aim to capture diversity in the young people’s experiences and navigation of various mental health related difficulties. Each of the videos have between three to seven lived experience speakers. To illustrate the type of lived experience videos created, one example of a participant transcript from an overall video compilation for each of the personal recovery themes will be described.

**Table 1:** Summary of the lived experience videos developed.

Module theme	Video title	Number of speakers	Content areas discussed
My Journey	My Journey	3	Brief overview of the speaker’s mental health experiences and meaning of “recovery”.
Self-Care	What is stress?	7	Things that can cause people stress (e.g., crowded places, financial instability, expectations from others).
	Impact of stress	6	The effect of stress including physiological responses, racing thoughts, or psychotic symptoms (e.g., increased voices).
	Managing stress	6	Strategies used to cope with stress such as distractive (e.g., exercise, meditation) and cognitive processing techniques.
My Identity	Who am I?	4	Sense of self and personal strengths (e.g., fitness, humour, religious beliefs, art).
	How my sense of self has changed	4	Impact of mental health on personal identity and coping with changes (e.g., acceptance, values identification, re-engaging with past interests).
	Stigma and discrimination	4	Experiences with stigma (e.g., societal and self-stigma) and how these situations were managed.
Connections	Relationships	4	The impact of mental health on relationships and navigating challenging relationships or loss of friendships, as well as how to form new reciprocal connections.
	Family	5	The impact of mental health on family systems (e.g., supportive responses, and navigating difficulties).
	Social media	5	Positive experiences with social media as a means of connecting and navigating the negativity that can arise.
Life	Challenges	3	Navigating different challenges in life (e.g., employment, finances, getting a driver’s license).
	Disclosure	5	Experiences with disclosing mental health to others (e.g., romantic partner, employment) and deciding when not to disclose.
Mental health	Experiences with mental health services	5	Experiences and difficulties with mental health services (e.g., engaging in service programs or relationship concerns with workers).
	Navigating difficulties with mental health services	5	Managing difficult situations such as conversations about medications, power imbalances or having multiple workers.

#### My Journey

The My Journey theme involves three participants briefly talking about some of their mental health experiences including a point in their journey where things started to get better. Two of the young people also describe their view on what “personal recovery” means to them to highlight the individualized nature of recovery. This was covered in a single video titled “My Journey”.

“How I started my mental health journey. At first, I did it by myself. I tried my luck so to speak. I used to get waves of depression, where six months I’d be feeling really depressed. Six months I’d be feeling fine, so I think, oh, that’s okay. But it did come back. I kept pushing through, gradually getting worse and worse each time, until I guess I reached a bit of a breaking point and I reached out and saw someone, which I regret taking so long to do. But kinda switched my views there. Started the long journey and now I feel like I’m the best possible me I can be at the moment. When I first got diagnosed, my view on recovery is a lot different to what my view is now. My view back then was this massive, I guess task, I had to do. I didn’t know if I was ever gonna reach recovery, it was quite blank and vague, the information given didn’t favour a recovery. But now my view has changed to seeing that recovery is about taking a lot of little small steps.” Interviewee 1.

#### Self-Care

The Self-Care theme involves young people talking about the various things that cause them stress, the impact of stress and the different ways in which they manage stress. The content covered in this theme was divided into three videos; what makes you stressed, the impact of stress, and managing stress. In the what makes you stressed video, seven participants spoke about the things or situations that can lead to them feeling stressed such as social situations, family relationships, studying, financial constraints, expectations from society, and a negative internal voice (i.e., “you’re not good enough”).

“The main thing I stress about is that I worry a lot. I worry about myself, how I’m going in life, how my friends are going in life, if anyone’s okay, if anyone’s ill. I worry a lot about my mental health.” Interviewee 2.

The second video focused on six participants describing the impact of stress on themselves. This included physiological, behavioral, and cognitive effects of stressors such as experiencing bodily pains, heart palpitations, shakes, and sweats, as well as changes to emotional reactions (e.g., feeling depressed or paranoid), or hearing increased voices.

“I get a lot of heart palpitations. My heart races, I get shakes and sweats. I get a bit queasy in the stomach and then I have thoughts racing that, you know “Why am I in this situations?” And you know it’s stressing me out, I need to get out of those situations because I don’t feel comfortable in those situations.” Interviewee 3.

The last video in the Self-Care theme involved six participants describing the different ways in which they manage stressors, and how these strategies are helpful for them. This included distraction-based strategies such as physical activity (i.e., going for a walk), practicing meditation or yoga, listening to music, as well as more cognitive-based strategies. These included time-management skills and exploring the nature of the stressor.

“I find that music can be therapeutic. Every time I’m stressed, I chuck some music on or chuck a beat on and I’ll write how I’m feeling or a big cure for me I turn to my faith and I pray.” Interviewee 4.

#### My Identity

The My Identity theme involves young people reflecting on their sense of self and how their view of themselves may have changed throughout their mental health experiences. My Identity also explores young people’s experiences with stigma and discrimination, and how they may have navigated such difficulties throughout their journey. The content covered in this theme was divided into three videos: who I am?, how my sense of self has changed, and navigating stigma and discrimination.

In the first video, four participants described their perspective on what makes them who they are and their personal strengths. There were differences across the perspectives, with one participant talking about the importance of religion and two participants describing the value of listening to and playing music, whilst another participant spoke about having strengths in socializing with others and humor. One participant recognized their sense of self encompasses a range of things including creativity and their mental health experiences.

“The things that make up who I am as a person? Oh, man. There are so many different things. I love color, flowers, art is the biggest one in there, though. I’ve been an artist ever since I was young, and it is every aspect of my life. Everything is a piece of art, and I love that. My mental health is actually a very big part of me, as well, I’ve come to realize. Obviously, when I was younger, I didn’t want it to be there at all, but I’ve come to realize that it’s a very, it makes me who I am, and without it, I wouldn’t be the stronger person I am today.” Interviewee 2.

The second video on how my sense of self has changed aimed to highlight four participants experiences associated with changes to their identity, whether that involved reconnecting with their sense of self before experiencing mental illness or forming a new sense of self following mental illness. The video captures what participants did to reconnect or shape their personal identity, such as embracing acceptance, identifying values, or re-engaging with prior interests or hobbies.

“After receiving the diagnosis, it definitely changed the way I thought of myself. I felt like I wasn’t me anymore. Currently, I feel a lot better. I feel that after a period of time, I definitely feel like I’ve better myself, more than anything. But at the time it was quite big and scary. The kind of things I did to find out who I am again after the diagnosis was just try to reconnect with what I was passionate with, before I started getting lost so to speak. Before I started throwing it all away. I started exercising, was one. Just really taking the time to think about what I wanted to do in life. Trial and error really. I did a lot of different courses to find out what I wanted to do. Did a lot of different jobs, and yeah, just really reconnected with my passions and let them blossom again.” Interviewee 1.

The navigating stigma and discrimination video covered both societal stigma and self-stigma. Capturing both aspects of stigma was important in being able to highlight the different types of stigma that can occur and obtain diversity in young people’s experiences. Two participants talked about experiencing societal stigma from people in the community or workplace, and two other participants described their experiences with self-stigma, and how they navigated these difficulties.

“The discrimination at work that was a real difficult one. A lot of the times in the workplace I felt like I wasn’t good enough or I’m off with the fairies. I’m just not normal. How I used to cope with discrimination of the people around me and my friends and everyone who didn’t want to be around me really. I didn’t cope at first but for a long time I was by myself. I didn’t know how to deal with what I was going through. I would turn to God a lot of the times. Having a new church to go to helped me cope with losing all my friends really but the discrimination at work that was a real difficult one. It didn’t get resolved. It got to the point where I had to walk away. I found that it was for the best to have a long break from everyone who was putting me down and not helping to then go off and find people that genuinely care.” Interviewee 4.

#### Connections

The Connections theme involves young people talking about the impact of mental health on their relationships with others in their physical and online environment, and how these relationships can be challenged. It also explores how people formed new relationships. The content covered in this theme was divided into three videos; relationships, family, and social media. In the relationships video, participants spoke about how their relationships with friends and the community might have changed throughout their mental health experiences, and how some people navigated losing friendships and forming new reciprocal connections with others.

“My mental health has impacted my family and it’s been difficult with friends as well, not understanding mental health or why someone does those type of things. They’re not sure how to help you or even talk to you sometimes about those situations or what you’ve dealt with. But over time people generally get a bit better. What I’ve done to feel more connected is basically I met a lot of people through the mental health sector and we kind of used mental health as a kind of, as grouping each other, that we all went through these different situations and we’ve all come together and we all mutually help each other. So, through that I’ve gained a lot of friendships and relationships and met a lot of great people just because we can all relate to each other.” Interviewee 3.

Another key area for young people was the impact of mental health on their family system. In the family video, participants described the difficulties experienced in feeling pressure from their parents to do well. Other participants spoke about a lack of understanding about mental illness from their parents, and how they navigated these difficulties. Positive experiences with family members were also described in the video.

“I remember when I started experiencing mental health difficulties, I remember quite a strong response which was a bit surprising for me. A strong response in that two of my older siblings lived back at home for a period of time to support my parents and everyone within the family and I found that really positive. When I was within the hospital system just my family making an effort in that my mother made sure that she came and saw me every day which was really important to know that I was getting that constant support and care.” Interviewee 5.

Social media was the third video within the Connections theme. The topic of social media was highlighted in the content development workshops as a key concern for young people beyond those discussed for relationships more broadly. In the video, participants shared both positive and negative experiences associated with various social media uses. Some participants spoke positively about social media in providing a sense of connectedness with others and sharing their own story, whilst other participants acknowledged the negativity that can occur from making a post online. One participant believed in the importance of communicating with others *via* traditional means of face-to-face or phone contact relative to social media.

“My view of social media is I don’t believe it has to be the only way to communicate with someone. The old way of phoning someone is still a prevalent way to talk to people and I have close friends myself, we keep in contact by phone and things like that. And face to face, as well instead of just through Facebook feeds and seeing what’s going on.” Interviewee *3*.

#### Life

The Life theme involves participants talking about the various challenges they have experienced in their life to date, and how their mental illness may have impacted on these challenges. This theme also involves participants talking about their experiences with disclosing their mental health to others, as well as participants describing situations where they decided not to disclose their mental illness. The content covered in this theme was divided into two videos; challenges and disclosure.

In the challenges video, three participants describe the different difficulties they have experienced and how they navigated these challenges. One participant spoke about the trouble experienced in obtaining a driver’s license whilst being on certain medications, and how they were able to overcome this issue through seeking support from their mental health worker. Another participant described feeling removed from society after experiencing their mental illness, and how they were able to navigate this difficulty through slowly re-connecting with activities. The third participant described difficulties associated with feeling scared about the possibility of relapse and finding the motivation to pursue employment.

“Some of the challenges I’ve had with mental health illnesses and I guess pushing forward in life is, the motivation to try to find work was hard, because it wasn’t quite there. I was very close off and I didn’t want to push forward. You know, don’t have any money from not working is quite stressful. You can’t go out and enjoy the time with your friends. You can’t do this, so you end up sitting at home, just being alone with your own thoughts. So, I was afraid to push forward, because I was afraid that I could relapse. I guess it took me a long time to get comfortable with pushing myself a little bit to find employment, to reach out for education, and to I guess help myself.” Interviewee 1.

The second video in the Life theme involved five participants talking about their positive and negative experiences with disclosing their mental illness to others such as with a romantic partner, friends, mental health professionals, and at work. It was important to capture both normalize and validate young people’s experiences in this area and highlight the difficulties that can occur. One participant spoke more broadly about only disclosing their mental health to people they trust and believe care about them.

“When I disclosed to my previous work about my mental health, it wasn’t very good that I felt like I was suddenly isolated by some people, and I found it very hard to connect with people, because the place that I worked, it was just, everyone talked to everyone, and everyone knew about everyone. The fact, I felt like people thought I’d been hiding something from them. That was pretty isolating, but I kind of realized that, although that had happened, it was okay, because my whole life wasn’t work, or wasn’t that work. Although, I’m not working there anymore, and I still have friends from there, I know that the people who didn’t quite like it, I guess, don’t matter because they are not a major part of my life”. Interviewee 2.

#### Mental Health

The Mental Health theme focuses on young people’s experiences with mental health services. This theme appeared to be vital in covering both the helpful and positive experiences some individuals have when engaging with mental health services, particularly for the first time, but also capturing content that highlights some of the difficulties faced by young people engaging in services such as navigating various key workers or power imbalances. The content for this theme is divided into two videos: experiences with mental health services and navigating difficulties with mental health services.

In the first video, five participants described their experiences with mental health services. Two participants described a positive and supportive experience, whilst another participant spoke about initially being frustrated but later finding value in receiving support form a professional who had specialized expertise. Another participant expressed wishing they had known more about mental health and the services available when they were unwell but described the opportunities now available for them such as group activities associated with the service. One participant highlighted the difficulty experienced when they first engaged with mental health services and spoke about how they managed the situation at the time, and in hind-sight what they would do differently based on their experience.

“My first contact with mental health services wasn’t the best. But I’ve had a lot of positive views, like, overtime, I’ve had a lot of positive experiences with mental health services as well. From my experience, the first person I saw wasn’t the best fit for me, but I wasn’t sure there was any other option out there, so I thought that was all I had. I was given this, could only do this. I was stuck at that for a while. What I wish I had have done was voiced my opinion and said, look, I don’t know if there are any other services that I could be seeing. Because I didn’t agree on some of his views, but I thought I was just stuck with that. It’s important to not just feel, be turned off your first experience, but to keep working, finding the one that fits you. It’s a bit of trial and error, but that’s with everyone.” Interviewee 1.

The second video of the Mental Health theme is navigating difficulties with mental health services. This video captured five participants experiences with mental health services including two participants experiences with understanding medication and talking about medication with their treating team, whilst another participant reflected on how they managed a power imbalance and a different participant spoke about managing having multiple workers from different organizations involved in their care. One participant described the value of having someone advocate for their treatment and support.

“When I first started using mental health services, I found that the most confusing part of it all was the medication. There were lots of different medications for different things, but I found that sometimes I was prescribed a medication that wasn’t mainly used for my illness, but was used for another illness, but I was being prescribed it, because apparently, sometimes it can help my illness. It all got a bit confusing and talking to mental health professionals or counsellors, workers, any sort of mental health person, about my medication has always been kind of hard for me” Interviewee 2

### Website Design

**Figure 3:**
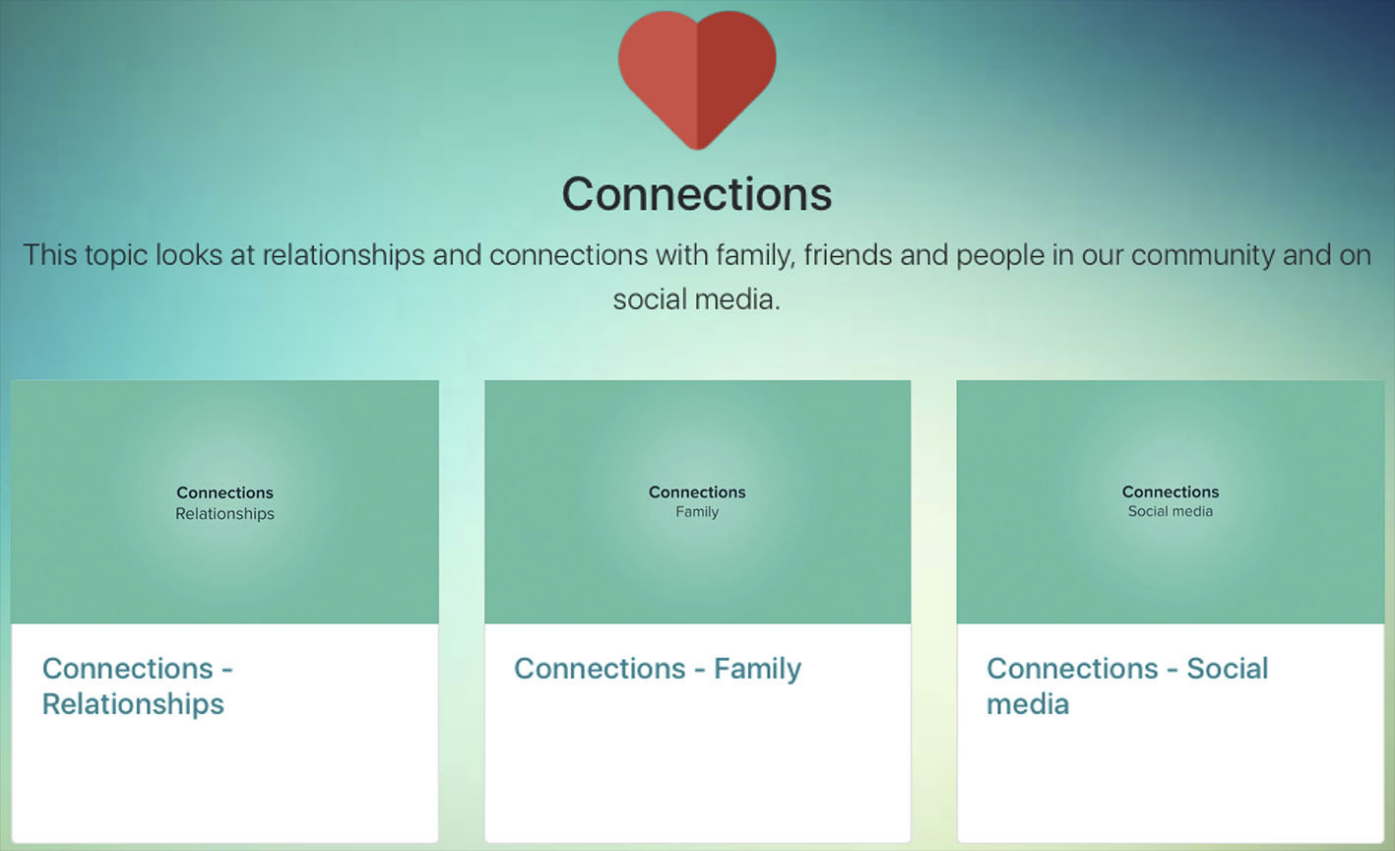
Example of the connections module in Peer Plus.

**Figure 4:**
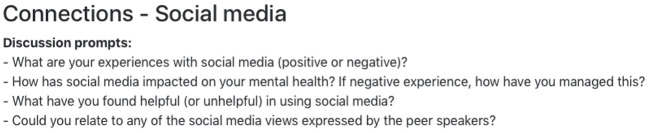
Example of the discussion prompts for the connections module in Peer Plus.

A simple website hosting the videos was professionally created in accordance to preferences expressed in the workshops (i.e., youth-friendly, engaging, easy to understand, and incorporate colors), PSWs preferences, and recommendations from the literature (64). The website employs a single page design optimized for a tablet computer (e.g., iPad), with simplified navigation by organising the content in the same format for all modules. Each module has an icon, heading, brief description, and easily accessible links to the lived experience videos (see **Figure 3**). By having the content on a single webpage, participants can easily scroll through the website to the personal recovery theme they would like to explore. Whilst the modules are presented in an order to allow for a single page format, participants are encouraged to choose the module themes and videos they would like to explore in the session. Discussion prompts are included with each video to help facilitate conversations, if required (see **Figure 4**). The website is accessed *via* a private account issued to the PSW before the individual face-to-face peer support work sessions. Considering the planned usage as a tool to stimulate discussion during peer work, rather than as a tool to be used outside sessions, young people were not issued with log-ons.

### Peer Plus Program

The development process resulted in the creation of a digitally-assisted personal recovery and self-management program for peer support work, namely, Peer Plus. As previously described, the program is based on six personal recovery modules that each contain a series of lived experience videos. Peer Plus is a four-session program designed to be used on an iPad by a PSW and young person in a face-to-face session to access the lived experience videos. Participants are introduced to the six personal recovery themes and decide what themes they would like to explore in each of the sessions. The lived experience videos are intended to help facilitate recovery-oriented conversations and promote a sense of hope and validation. The next phase of this project involves a pilot trial of Peer Plus across the Alfred hYEPP sites in Victoria, Australia. The feasibility, acceptability and preliminary outcomes of Peer Plus in young people aged 16–25 years old is currently being investigated. Formal pilot trial data will be provided in detail in future reports and publications.

## Discussion

In this paper, we illustrated how a novel digital video-based peer support resource could be developed through a participatory process. These early research processes are more commonly being documented given the benefits in disseminating knowledge and experience in developing new methods (65).

The detailed descriptions of the personal recovery themes and lived experience video development showcases the ways in which differing expertise can be involved in the creation of new tools (65). The process of transforming consumer material from the content development workshops into a series of digital videos was an innovative approach to creating peer resources that were grounded in lived experience. Illustrating this approach helps create dialogue on how digital technology could be integrated into the peer support work context and used to establish meaningful resources (29). Ultimately, by valuing and incorporating consumers, mental health service, and PSWs perspectives throughout the study, we were able to develop personal recovery based lived experience videos as part of a broader digitally assisted peer support program. This participatory process may assist in breaking down some of the barriers commonly associated with translating research into practice and provide a program that may be utilized by the peer workforce (58, 66).

The development process described here substantially refines the video production process developed in the SMART research program (31) by generating the initial content framework *via* a participatory process, and embedding user participation at all steps of development. In this way content was developed *via* a relatively pure “bottom-up” process of synthesising lived experience content from an initial consumer-based framework, rather than a “top-down” process in which content is based on a framework developed by researchers or developers. This bottom-up approach had particular application within the explicit lived experience-based domains of personal recovery and peer work, and was especially suitable given the lack of existing models of personal recovery for young people experiencing psychosis at the time of development. More top-down aspects may nonetheless be valuable in other applications where there is an existing therapeutic model to translate. For example, developing video material for a self-guided program for bipolar disorder (67, 68) has involved developing an initial framework based on empirically-supported treatment models, and using lived experience input to inform how to communicate these concepts. Understanding how personal recovery could be conceptualized in young people experiencing psychosis was important in learning about the specific personal recovery priorities relevant to this population to create the digital peer videos. Existing research on the theoretical framework of personal recovery in mental health [i.e., CHIME; (11)] have predominantly been conducted in adult mental health populations, with the exception of a couple of studies exploring personal recovery in young people with a range of mental health experiences (17, 69). The specific personal recovery areas for young people with psychosis, however needs to be explored further, as personal recovery is a dynamic process (59).

The six recovery themes that arose from the development process drew similarities with previous research [i.e., (11, 16, 17, 70)]. There was substantial overlap with the CHIME framework, but a number of new areas arose, and common areas were reframed to align more closely with young people. For example, the connections and my identity themes appeared to be consistent with the connectedness and identity process in CHIME (11). The mental health theme involved conversations about young people’s experiences and challenges with mental health services, and how such challenges were navigated, which drew parallels with empowerment in CHIME (11), as well as Bird et al. (12) issues around misdiagnosis and medications. Similarly, life appeared to share similar underlying themes with the meaning in life and empowerment processes of CHIME, as well as Bird et al. (12) practical support theme. The CHIME process of hope and optimism for the future appeared to align with my journey but also presented as being important across all themes. The young people sharing their own experiences and navigation of difficulties in the videos aimed to create a sense of hope and belief in the possibility of personal recovery.

A new area that arose within the realm of connections was participants preference to hear lived experience perspectives on the impact of social media. Research has shown young people with psychosis are increasingly using digital technology (28), in which this may be a topic relevant to a youth population in connecting with others. Additional differences arose in the various life challenges associated with the age and development of young people (e.g., getting a driving license while on certain medication). This highlights the necessity of integrating lived experience accounts into research to prioritize relevant recovery factors (59, 69). However, it is important to note the purpose of the current study and development of the recovery-oriented themes was to inform the creation of the digital tool. These themes arose from a small sample size, which limits the generalizability of these findings. Research is required to more thoroughly investigate the personal recovery themes specific to young people experiencing psychosis.

Although successfully creating a digital resource, challenges occurred throughout the development process. There was difficulty experienced in identifying young people who were willing to share their lived experiences on film. The video development phase was extended six months in an attempt to capture additional content and diversity in the people filmed, however a decision was made to include additional interview material from another resource [i.e., (31)]. This allowed for 10 lived experience speakers to be in the videos, which provided diversity in age, gender, sexuality, and ethnicity. An adequate amount of detail was obtained to broadly cover each of the personal recovery themes, however not all of the areas had sufficient content to generate viable interview material for the videos, in which these areas were revised based on the content obtained.

Additional time and practical constraints associated with mental health service staff turnover lengthened the development stage and led to difficulty in maintaining lived experience involvement in the entire development process. For instance, there was a total of two team leaders, three service professionals, and nine PSWs involved at differing time-points throughout the study, which led to time being spent developing new relationships with the employees, orientating them to the project and discussing potential changes to tailor the program to the new PSWs preferences and model of working with young people to improve implementation. These changes, alongside the extensive and time-consuming nature of the video development process also meant it was not feasible to have lived experience input in all phases of the video development, in which the researchers own biases may have influenced the final video clips included in the videos. Lastly, while the videos were designed to contrast a variety of experiences related to mental illness and personal recovery, it is important to note that the researchers endeavoured to create an overall positive feel to the videos, which could have minimized some of the difficulties experienced with mental illness (71).

The model described has potential to develop analogous tools and models of working for other populations. A similar digital tool has been developed for adults with severe and enduring mental health problems (31), but this has yet to be trialled as a peer work tool. There may be additional applications to other settings in which PSWs are employed such as peer group programs. It is notable that the use of this tool requires interest from both consumers and PSWs, which may limit uptake. Non-peer workers have expressed some support for digital interventions in mental health services, but identified key barriers related to their use and implementation [e.g., accessibility and usefulness to service delivery; (33, 72)]. Further research is needed to understand the views of PSWs who were not involved in the development process on the use of digital tools in peer work.

In conclusion, the study findings highlight a new avenue for the development of digital tools in peer support work and beyond. The contributions made by consumers, mental health service, and PSWs were instrumental in being able to create digital resources that may be more likely to be tailored to young people experiencing psychosis and utilized by the peer workforce (66, 73). Such understanding would not have been achieved without their involvement. The use of personal recovery based lived experience videos as a tool for change by facilitating conversations about a young person’s own mental health experiences and promoting a sense of hope within peer support work is an area to be investigated.

## Data Availability Statement

Within the bounds of participant consent, the datasets generated for this study are available on reasonable request to the corresponding author.

## Ethics Statement

The studies involving human participants were reviewed and approved by Swinburne University of Technology and The Alfred Human Research Ethics Committee. The participants provided their written informed consent to participate in this study. Written informed consent was obtained from the individual(s) for the publication of any potentially identifiable images or data included in this article.

## Author Contributions

All authors were involved in conceptualizing, planning, and developing the peer support intervention model and digital tool. All authors contributed to the article and approved the final version.

## Funding

The project was funded by the Barbara Dicker Brain Sciences Foundation Grant (awarded to CP, ML, and NT), Alfred Health, and an Australian Government Research Training Program scholarship (awarded to CP).

## Conflict of Interest

The authors declare that the research was conducted in the absence of any commercial or financial relationships that could be construed as a potential conflict of interest.
